# Supplementary material for: A novel experimental design approach to generating orbital angular momentum waves using wearable textile antenna for sub-6 GHz 5G
Source: PLoS One. 2025 May 30;20(5):e0310113. doi: 10.1371/journal.pone.0310113 (PMC12124574; doi:10.1371/journal.pone.0310113)
Supplement: Attachment — Submitted filename: Response To Reviewers.pdf [file pone.0310113.s004.pdf]

Authors response

Reviewer 2

Comment 1) The proposed novel experimental design approach mentioned in the title should be briefly elaborated in the abstract section.

Authors response: Thank you for your comment. We have added a new line in the abstract section.

2) The advantages of the proposed novel experimental design approach can be highlighted in the abstract section.

Authors response: Thank you for highlighting this issue. We have mentioned the advantages of the proposed design in the revised version.

3) The dimensions of the single elements and array antennas should be mentioned in the abstract section

Authors response: Thank you for your constructive comment. We have added the dimension of the array and each element in the abstract of the revised version.

4) Metamaterials and metasurfaces are interesting methods to realize high performance antennas, so to improve the quality of the introduction section authors can add a short paragraph including some information about these techniques. Below are helpful suggestions.

Authors response: Thank you for the helpful suggestion. To generate OAM waves, compared to metasurface, patch array antenna is more matured approach as mentioned by the authors in" S. K. Noor et al., "A Review of Orbital Angular Momentum Vortex Waves for the Next Generation Wireless Communications," in IEEE Access, vol. 10, pp. 89465-89484, 2022, doi: 10.1109/ACCESS.2022.3197653.". To generate OAM waves, metasurface tends to be bigger in size with complex unit cell designs. As wearable and body worn antenna is concerned, it is preferred to keep the design small and simple.

5) At both sides of the feeding lines of the radiation patches, authors have made two cutes, please explain the impact of these cutes?

Authors response: Thank you for the comment. This method of cutting both sides of the radiating patch is called "Inset feed" technique. Inset Feed causes high impedance matching so that maximum power from the generator side reaches near the center of the patch. We have mentioned the reason in Section 2.1 of the revised version.

6) The meander lines connecting the feed lines of the upper patches are different from the meander lines connecting the feed line of the bottom patches, please explain why these meander lines connecting the patches to the main feed line do not have the same configuration? In other words, why are they not symmetric?

Authors response: Thank you for the constructive comment. As it can be seen from Figure 1 (a) that the meander lines connecting to Patches 3 and 4 are the mirror image of the meander line connecting to the Patches 1 and 2. Hence, the same feeding network of patches 1 and 2 was used for patches 3 and 4 which gave a 180° phase rotation. To generate OAM mode 1, the phase difference between the each element should be 90° and from patch 1 to patch 4 will be in the sequence of 0°, 90°, 180° and 270°. Our obtained result is very close with the theoretical design as it can be seen from Figure 2a. The phase increment from patch 1 to patch 4 of our design is 2°, 91°, 182° and 273° with maximum percentage error 1.11%.

7) How have authors addressed the mutual coupling between the radiation patches?

Authors response: Thank you for highlighting this concern. To avoid mutual coupling between the radiating elements to generate OAM waves, the spacing between each element should be atleast 0.7λ to avoid mutual coupling as mentioned in

Qiang Bai, A. Tennant, B. Allen and M. U. Rehman, "Generation of orbital angular momentum (OAM) radio beams with phased patch array," 2013 Loughborough Antennas & Propagation Conference (LAPC), 2013, pp. 410-413.

We have mentioned about how we avoided mutual coupling of our design in Section 2.1 of the revised paper.

8) Looking at the provided figures, it seems authors have bent the array antenna in one direction (vertical direction), did authors have bent it in another direction (horizontal) as well?

Authors response: Thank you for your comment regarding this issue. The array antenna is bent across vertically which is equivalent to bending each radiating patch horizontal as you can see from Figure 4. It is because the vertical side of the radiating element has major effect on operating frequency. To make sure the bending doesn't cause the antenna to operate in other frequency rather than the desired frequency, the bending is carried across horizontal side of the radiating elements which is vertically of the array antenna.

9. Besides Fig. 11 (b) please add the 2D radiation efficiency plot as well.

Authors response: Thank you for your comment. This paper focused on gain, mode purity and bandwidth as of now for airgap concept. The use of airgap effect on radiation efficiency is not the scope of this project however we are at the moment extensively studying the effect of airgap on radiation efficiency, directivity and power loss for a different project.

10. Please support the conclusion with more numerical results

Authors response: Thank you for highlighting this concern. We have revised the conclusion part in the revised version by adding airgap results and SAR results.

Reviewer 3

1. The introduction does not provide a comprehensive overview of the topic, and there are insufficient references. For this kind of paper, 25 references used in the paper are not sufficient.compare you results with other papers covering OAM ,5G NR Band and Enhansing Gain article like.

Authors response: Thank you for your valuable comment. The main objective of this paper is “OAM waves using flexible textile substrate”. Out of the two papers you mentioned, the first paper with the title “S. K. Noor, A. M. Ismail, M. N. M. Yasin, M. N. Osman, N. Ramli et al., "Generation of OAM waves and analysis of mode purity for 5g sub-6 ghz applications," Computers, Materials & Continua, vol. 74, no.1, pp. 2239–2259, 2023.” Is our paper where we generated OAM waves with hard FR-4 substrate. We have cited this paper in [20] . In the revised version, we have compared our this paper with Ref [20] in table 4

4.Gramatical mistake are too many,need to improve english abit specially in Methodology and Result analysis section.

Authors response: Thank you for highlighting this issue. We have corrected the errors in the revised version.

5. The Conclusion part of the paper is too short and the deeper scientific discussion about the achieved results is missing.

Authors response: Thank you for your constructive comment. We have revised the conclusion paper in the revised version with scientific discussion and numerical results.

Reviewer 4

1. The background of the research should be given in a separate section. Also, use other up-to-date and valid articles. Finally, at the end of this section, a category of existing methods for designing, increasing gain, increasing bandwidth, and reducing dimensions will be presented.

Authors response: Thank you for your valuable comment. The proposed work is first of its kind where OAM waves is generated using wearable flexible textile material. Therefore, there is no existing paper which has carried out research in this topic to the best of our knowledge. That is why, there is a limitation in comparing the achieved results with previous works. However, as tabulated in Table 4, the results of this paper is compared with other papers in terms of design complexity, mode purity, bandwidth and SAR analysis.

2. For flexible dielectric, it is mandatory to provide SEM images.

Authors response: Thank you for your comment. The Scanning Electron Microscope (SEM) is basically important in a paper where the material is a novel material. In this paper, we used Felt textile substrate with relative permittivity of 1.44 and thickness 3 mm. The description of the substrate is shown in the figure below.

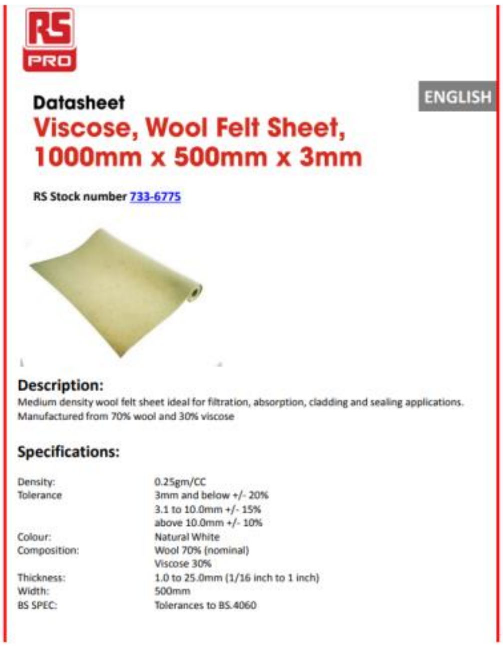

RS PRO

ENGLISH

Datasheet

Viscose, Wool Felt Sheet, 1000mm x 500mm x 3mm

RS Stock number 733-6775

Description:

Medium density wool felt sheet ideal for filtration, absorption, cladding and sealing applications. Manufactured from 70% wool and 30% viscose

Specifications:

Density:	0.25gm/CC
Tolerance	3mm and below +/- 20%<br>3.1 to 10.0mm +/- 15%<br>above 10.0mm +/- 10%
Colour:	Natural White
Composition:	Wool 70% (nominal)<br>Viscose 30%
Thickness:	1.0 to 25.0mm (1/16 inch to 1 inch)
Width:	500mm
BS SPEC:	Tolerances to BS.4060

3. Although a series of parametric reports have been presented, it is necessary to investigate the effect of changes in the type of dielectric (felt, jeans, cotton, etc.), change in dimensions, and change in dielectric thickness on each of the characteristics of S11, realized gain, radiation patterns at central frequency, and radiation efficiency.

Authors response: Thank you for your constructive comment. The main focus of this paper was to generate OAM waves experimentally which has been successfully done. In addition, since flexible substrate was used hence the effect of bending or deformation on OAM characteristics has been presented in the paper. The parametric analysis mentioned in the comment can be used for a different project focusing only the analysis part. We are currently working on a similar project to investigate the above mentioned things. Hence, we really appreciate the feedback given by the reviewer
