# Supplementary material for: A novel experimental design approach to generating orbital angular momentum waves using wearable textile antenna for sub-6 GHz 5G
Source: PLoS One. 2025 May 30;20(5):e0310113. doi: 10.1371/journal.pone.0310113 (PMC12124574; doi:10.1371/journal.pone.0310113)
Supplement: Attachment — Submitted filename: PLOS_revision.pdf [file pone.0310113.s003.pdf]

Authors have addressed most of the queries raised by various reviewers in appreciable manner. However, there are few queries from my end and hope authors shall address them. The manuscript can be accepted for publication with minor revision.

Comments:

#1. There is no mention about the polarization of the proposed design in the manuscript.
#2. There are several grammatical errors in the manuscript.
#3. The authors in the manuscript don't mention whether the results like radiation pattern, gain, bandwidth etc. achieved are on-body or free space results. And if there is any difference between the two the authors are encouraged to showcase the same.
#4. Is there any effect of body on the generation of OAM waves when the antenna is mounted on the body? And if yes, discuss in the manuscript.
