# Supplementary material for: A novel experimental design approach to generating orbital angular momentum waves using wearable textile antenna for sub-6 GHz 5G
Source: PLoS One. 2025 May 30;20(5):e0310113. doi: 10.1371/journal.pone.0310113 (PMC12124574; doi:10.1371/journal.pone.0310113)
Supplement: Attachment — Submitted filename: Response to Reviewers.docx [file pone.0310113.s002.docx]

Original Manuscript ID: PONE-D-23-04350

Original Article Title: A novel experimental design approach to generating OAM waves and analysis of airgap technique for gain enhancement in flexible and wearable antennas

To: PLOS ONE Editors

Re: Response to reviewers

Dear Editor,

Thank you for allowing a resubmission of our manuscript with revised version, with an opportunity to address the reviewers’ comments. We really appreciate your timely handling of our manuscript and the excellent review process. After revising the manuscript based on the Reviewers comments that we have received, we would like to submit the revised manuscript to be considered for publications in PLOS ONE. In the revision, we have addressed all the comments and suggestions given by the Editor and the Reviewers. For convenience, the response to the reviewers are written and it is in bold fonts. The changes made in the revised version of our manuscript are detailed in the point-by point response to the Reviewers comments. Lastly, we would like to thank the Editor and the Reviewers of PLOS ONE, whose efforts have significantly improved the quality of the paper. We are uploading (a) our point-by-point response to the comments (below) (response to reviewers), (b) an updated manuscript with yellow highlighting indicating changes (Revised Manuscript with Track Changes), and (c) a clean updated manuscript without highlights (Manuscript).

Best regards,

Shehab Khan Noor

**Response to Reviewers**

Reviewer’s comment 1

Is the manuscript technically sound, and do the data support the conclusions?
The manuscript must describe a technically sound piece of scientific research with data that supports the conclusions. Experiments must have been conducted rigorously, with appropriate controls, replication, and sample sizes. The conclusions must be drawn appropriately based on the data presented.

Reviewer #1: Yes

Reviewer #2: Yes

**Response to reviewer’s comment: Thank you for evaluating our paper. We appreciate the fact that both the reviewers found our work technically sound.**

Reviewer’s comment 2

Has the statistical analysis been performed appropriately and rigorously?

Reviewer #1: N/A

Reviewer #2: Yes

**Response to reviewer’s comment: Thank you for your valuable comments.**

3. Have the authors made all data underlying the findings in their manuscript fully available?

The [PLOS Data policy](http://www.plosone.org/static/policies.action#sharing) requires authors to make all data underlying the findings described in their manuscript fully available without restriction, with rare exception (please refer to the Data Availability Statement in the manuscript PDF file). The data should be provided as part of the manuscript or its supporting information, or deposited to a public repository. For example, in addition to summary statistics, the data points behind means, medians and variance measures should be available. If there are restrictions on publicly sharing data—e.g. participant privacy or use of data from a third party—those must be specified.

Reviewer #1: Yes

Reviewer #2: Yes

**Response to reviewer’s comment: Thank you for your comments and we certainly appreciate the positive response of the reviewers regarding the data of our manuscript.**

4. Is the manuscript presented in an intelligible fashion and written in standard English?

PLOS ONE does not copyedit accepted manuscripts, so the language in submitted articles must be clear, correct, and unambiguous. Any typographical or grammatical errors should be corrected at revision, so please note any specific errors here.

Reviewer #1: Yes

Reviewer #2: Yes

**Response to reviewer’s comment: Thank you for your positive response regarding the English standard of our manuscript.**

5. Please use the space provided to explain your answers to the questions above. You may also include additional comments for the author, including concerns about dual publication, research ethics, or publication ethics. (Please upload your review as an attachment if it exceeds 20,000 characters)

Reviewer #1: This article presents a way of designing wearable antenna. It is presented in well organized manner and many issues related to wearable antennas have been addressed. However, following points need to be considered before its acceptance for publications.

1. Almost all figures are too hazy.

**Response to Reviewer: Thank you for your constructive suggestion. We do apologize that the figures were not very clear. However, we have updated the quality of the figures in our revised paper.**

2. Review PDF is not properly created, Unable to find the referred figures from the text.

**Response to Reviewer: Thank you for highlighting this issue. We have updated the file containing the figures with proper figure as per as the manuscript.**

3. Gain value shown here, is measured or simulated? If it is simulated then it has to verified by measured results and vice versa.

**Response to Reviewer: Thank you for your valuable comment. The simulated gain is 3.762 dBi and the measured gain is 3.18 dBi. We have carried out the simulation as well as measured the gain of the proposed wearable antenna. We have verified both the simulated and measured gain of the proposed antenna. We have highlighted this part in the abstract as well as section 3.3.**

4. What about the efficiency of the antenna?

**Response to Reviewer: The efficiency of the proposed antenna is 17%.**

**1. As the number of radiating elements increases, the gain increases however the efficiency decreases. For one radiating element, the gain obtained was 3.32 dBi with efficiency of 34.72%. Next, when two radiating elements were used, the gain increased to 6.164 dBi however the efficiency reduced to 30.3%. Lastly, when four radiating elements were used, the gain increased to 8.109 dBi nevertheless the efficiency further reduced to 21.2%. In this paper, the substrate used was felt textile with permittivity of 1.44 , loss tangent of 0.044 and thickness of 1.5 mm to operate at 5.35 GHz ( H. A. Mashaghba et al., "1×4 Patch Array All-Textile Antenna for WLAN Applications," 2020 2nd International Conference on Broadband Communications, Wireless Sensors and Powering (BCWSP), Yogyakarta, Indonesia, 2020, pp. 21-25, doi: 10.1109/BCWSP50066.2020.9249401.)**

**2. The type of antenna used in this paper was microstrip patch antenna. Compared to other types of antenna, microstrip patch antennas tend to have lower gain efficiencies (Mousaab M. Nahas and Mouaaz Nahas “Bandwidth and Efficiency Enhancement of Rectangular Patch Antenna for SHF Applications” Eng. Technol. Appl. Sci. Res. Vol.9, No.6, 2019, 4962-4967.**

**3. The loss tangent of the felt substrate we used was 0.02 which is quite high. This is another possible reason behind low efficiency. Textile materials with high losses results to lower efficiency of the antenna ( A. Alomainy and T. H. Loh, "Experimental evaluation of wearable antenna efficiency for applications in body-centric wireless networks," 2014 IEEE MTT-S International Microwave Workshop Series on RF and Wireless Technologies for Biomedical and Healthcare Applications (IMWS-Bio2014), London, UK, 2014, pp. 1-3, doi: 10.1109/IMWS-BIO.2014.7032454.)**

**From the simulation as shown in below, the total efficiency and radiation efficiency is almost the same at 3.5 GHz (desired frequency). The radiation efficiency is -7.319 dB and total efficiency is -7.729 dB at 3.5 GHz. Since the difference is very less hence it indicates that the antenna matched well. There are several other methods that can be used to increase the antenna gain and efficiency such as airgap technique and electromagnetic band gap (EBG) method. Future researchers who would be working on wearable antenna for OAM waves generation can work on this part of the research to improve antenna gain and efficiency.**

5. There are many wearable antenna available in the market? Where the proposed antenna finds novelty?

**Response to Reviewer: Thank you for your valuable comment. The proposed antenna is first of its kind to generate OAM waves where a flexible and wearable antenna was used to generate OAM waves. The other antennas in the previously published paper and in the market mainly focused on generate Plane Electromagnetic Waves based wearable antenna, only single transmission and receiving signals can be exchanged. Moreover, in this manuscript, a novel study on the effect of antenna bending on OAM waves characteristics such as phase distribution, intensity distribution, mode purity and polar form was carried out. Lastly, to the best of our knowledge, we implemented the concept of airgap technique in flexible and wearable antenna for gain enhancement for the very first time. It has been noticed that, the concept works well and it increased the gain of the antenna (Simulation studies) from 3.762 dBi to 5.327 dBi with 1 mm airgap thickness without significantly affecting the OAM mode purity. As the demand for body centric devices are increasing exponentially, hence the proposed antenna can be used to transmit and receive multiple data from multiple devices using different OAM mode numbers using single antenna. An OAM wave theoretically has an infinite number of orthogonal modes that are orthogonal to one another. By multiplexing different OAM modes in the same frequency channel, this orthogonality feature presents a unique opportunity to improve spectral efficiency for 5G technology . Furthermore, OAM provides a new "Mode Division Multiple Access" scheme where different mode numbers can be assigned to different users within the same frequency without interfering with one another and without consuming additional resources such as frequency and time . In this way, large amounts of data from different users can be transmitted simultaneously without degrading the system's overall performance in body centric communication systems.**

6. Pls keep a dimension comparison in the comparison table. Mind that your proposed antenna has a large dimension (170 mm x 156 mm)

**Response to Reviewer: Thank you for highlighting this issue. We have updated Table 4 in the revised version of the manuscript where we compared the dimension of the proposed antenna with other recently published work’s dimension. Since the dimension of the antenna depends on the working frequency hence the comparison is made in terms of lamda (λ_0_) where λ_0_ is found by dividing the speed of light with the operating frequency of the antenna**

7. How the airgap between substrate and ground plane been created physically for increasing gain?

**Response to Reviewer: The airgap in the simulation can be created by “vacuum” where the dielectric constant value is 1. In practical, either a spacer or a foam with dielectric constant value of 1 can be placed in between the substrate and ground plane. By increasing the gap or distance between the substrate and ground plane, we are basically increasing the thickness of the substrate. The thickness of the substrate or dielectric material determines the magnitude of radiation of electromagnetic wave. Hence, if the thickness of the substrate is increased to a certain point, the dielectric constant of the radiating plane as well as the electric field concentration on the lossy epoxy is reduced which eventually enhances the antenna gain.**

8. How the antenna structure following UCA pattern? mention in the introduction part.

**Response to Reviewer: Thank you for your valuable comment. We have revised the paper based on the reviewers comment. We have added statement in the introduction part briefly to explain the UCA pattern of the antenna.**

Reviewer #2: This research work has presented and realized a wearable antenna to generate orbital angular momentum waves with mode +1. Promising results have been achieved and well discussed in the well-organized manuscript. So, the results have been experimentally validated and highlighted by providing a fair comparison with state-of-the-art. Although the concept and idea of this work were found interesting and they seem attractive for the scientific society, authors are requested to carefully address the following comments to improve the quality of the manuscript prior to final recommendation:

1) The design process of the proposed wearable textile array antenna can be briefly elaborated in the abstract section. Since this is an array antenna, please explain how authors have addressed the mutual coupling issue between the radiation elements in the proposed structure?

**Response to Reviewer: Thank you highlighting this issue. In order to generate OAM waves using uniform circular array (UCA) antenna, the minimum spacing between the radiating elements must be atleast 0.6λ as mentioned in the paper “Experimental circular phased array for generating OAM radio beams” Electronic letters, pg: 1414 -1415, volume 50, issue 20. In our design, the spacing between the radiating elements is 128 mm which is equivalent to 1.48 λ. As 1.48 λ > 0.6λ, hence we can make sure that the mutual coupling issue did not affect results of the proposed antenna. We have also added a statement in section 2 in the revised paper as per as the authors comment.**

2) Please highlight advantages of the proposed wearable textile array antenna in the abstract section.

**Response to Reviewer: Thank you for your valuable comment. We have addressed this comment and revised the abstract of the manuscript in the revised version.**

3) Introduction section can be improved by adding more comprehensive discussions along with suitable references. For example, mutual coupling reduction is an important issue to realize an array antenna. There are various decoupling methods in the literature which can be briefly elaborated in the introduction section along with proper references. Below are helpful suggestions.

**Response to Reviewer: Thank you for valuable suggestions and we really appreciate it. The papers suggested by the reviewer focus on Metamaterials and terahertz frequency range (VHF). The antenna proposed in this paper is based on the concept of microstrip patch antenna. And the frequency used is 3.5 GHz. Hence, the papers recommended by the reviewer don’t align well with our manuscript. However, in the future, while working in metamaterials, we shall definitely use the recommended papers.**

4) The array structure is constructed of 4 radiation elements, please explain why authors have implemented two cuts in each radiation element close to their feeding part? All the radiation elements are connected to each other by meander lines, please describe how authors have optimized these meander lines? (from their size point of view and from their topology point of view).

**Response to reviewers: Thank you so much for highlighting this. We really appreciate it. The two cuts in each radiation elements is called “Inset Fed Technique”. For conventional patch antenna, the radiating elements is excited by the centre feeding method. The conventional centre feeding technique is replaced by inset feeding because the current is low at the ends of a half-wave patch and increases in magnitude toward the centre.The feeding network is based on T-junction feed network to make sure equal power (amplitude) has arrived at each of the output ports.** **The combination of 50 ohm and 100 ohm feeding network was used to make sure that each radiating elements is receiving equal or almost same power amplitude to generate OAM waves with desired mode number.**

5) The feeding mechanism of the array should be elaborated in depth.

**Response to reviewers comment: Thank you for your valuable comment. We have revised the paper based on this comment in Section 2.1 of the manuscript**

6) Quality of the figures need to be improved.

**Response to reviewers comment: Thank you for your valuable comment. We have improved the figures in the revised paper.**

7) Can authors add the 2D curves of the radiation gain and efficiency before and after vertical and horizontal bending to compare the effect of bending on the radiation properties?

**Response to Reviewer: Thank you for highlighting this issue. Moreover, we really appreciate your efforts for pointing this issue. In section 3.6, we have mentioned that the bending is carried out across y-axis. It is because the y-axis of the proposed antenna is the width of the antenna while x-axis is the length of the proposed antenna. The length of the antenna has major influence in antenna’s operating frequency as per the authors in the following papers:**

**[1] L. Song and Y. Rahmat-Samii, "A Systematic Investigation of Rectangular Patch Antenna Bending Effects for Wearable Applications," in IEEE Transactions on Antennas and Propagation, vol. 66, no. 5, pp. 2219-2228, May 2018**

**[2] S. K. Noor, N. Ramli, N. H. A. Rahman and M. Najib Mohd Yasin, "Performance Comparison between Polyester and Denim Textile Antenna on Non-Identical Human Upper Arm Sizes for 5G mid-band," 2021 7th International Conference on Space Science and Communication (IconSpace), Selangor, Malaysia, 2021, pp. 40-45.**

**To make sure the antenna operates within its desired frequency range even after bending, we avoided the antenna bending across x-axis. In addition, the main objective of this paper was to study the effect of antenna bending on OAM’s four characteristics. In Table 3, we comprehensively studied the effect of bending on the four characteristics of OAM (phase distribution, intensity distribution, mode purity and polar form).**

8) In the comparison table 4, please add the term “design complexity” as well.

**Response to Reviewer: Thank you for your valuable comment. We have updated the comparison table as per as the reviewer’s comment.**

6. PLOS authors have the option to publish the peer review history of their article ([what does this mean?](https://journals.plos.org/plosone/s/editorial-and-peer-review-process#loc-peer-review-history)). If published, this will include your full peer review and any attached files.

If you choose “no”, your identity will remain anonymous but your review may still be made public.


**Do you want your identity to be public for this peer review?** For information about this choice, including consent withdrawal, please see our [Privacy Policy](https://www.plos.org/privacy-policy).

Reviewer #1: **Yes:** Saumya Das

Reviewer #2: No
