# Supplementary material for: A novel experimental design approach to generating orbital angular momentum waves using wearable textile antenna for sub-6 GHz 5G
Source: PLoS One. 2025 May 30;20(5):e0310113. doi: 10.1371/journal.pone.0310113 (PMC12124574; doi:10.1371/journal.pone.0310113)
Supplement: Attachment — Submitted filename: Response_to_reviewers_auresp_3.docx [file pone.0310113.s005.docx]

Original Manuscript ID: PONE-D-23-04350

Original Article Title: A novel experimental design approach to generating orbital angular momentum waves using wearable textile antenna for sub-6 GHz 5G

To: PLOS ONE Editors

Re: Response to reviewers

Dear Editor,

Thank you for allowing a resubmission of our manuscript with revised version, with an opportunity to address the reviewers’ comments. We really appreciate your timely handling of our manuscript and the excellent review process. After revising the manuscript based on the Reviewers comments that we have received, we would like to submit the revised manuscript to be considered for publications in PLOS ONE. In the revision, we have addressed all the comments and suggestions given by the Editor and the Reviewers. For convenience, the response to the reviewers are written and it is in bold fonts. The changes made in the revised version of our manuscript are detailed in the point-by point response to the Reviewers comments. Lastly, we would like to thank the Editor and the Reviewers of PLOS ONE, whose efforts have significantly improved the quality of the paper. We are uploading (a) our point-by-point response to the comments (below) (response to reviewers), (b) an updated manuscript with yellow highlighting indicating changes (Revised Manuscript with Track Changes), and (c) a clean updated manuscript without highlights (Manuscript).

Best regards,

Shehab Khan Noor

**Authors response**

**Reviewer #2:**

**Comment 1: In the abstract section please briefly mention the design process of the proposed wearable textile array antenna?**

**Authors response:** Thank you for your comment. We have added a new line in the abstract section.

**“**The overall dimension of the array antenna is 170 × 156 mm^2^ while the dimension of each element is 35 × 35.7 mm^2^. In order to control the phase and generate OAM waves, the proposed antenna was designed using a felt textile substrate and meandering lines of various lengths connecting the radiating patches.”

**Comment 2: In the abstract section please mention how the isolation between the array elements have been addressed?**

**Authors response:** Thank you for your constructive comment. We have added the information about the isolation/separation between the elements to avoid mutual coupling among the array elements in the abstract of the revised version.

**Comment 3: In the abstract section please mention how much is the distance between the radiation elements?**

**Authors response:** Thank you for you comment. We have mentioned about the distance between the radiation elements in the revised version. The distance was 1.48λ. Thank you for highlighting this concern. To avoid mutual coupling between the radiating elements to generate OAM waves, the spacing between each element should be atleast 0.7λ to avoid mutual coupling as mentioned in:

Qiang Bai, A. Tennant, B. Allen and M. U. Rehman, "Generation of orbital angular momentum (OAM) radio beams with phased patch array," 2013 Loughborough Antennas & Propagation Conference (LAPC), 2013, pp. 410-413.

“1.48λ was the separation between radiating patches in order to prevent mutual coupling between them”

**Comment 4: Advantages of the proposed wearable textile array antenna should be highlighted in the abstract section.**

**Authors response:** Thank you for highlighting this issue. We have mentioned the advantages of the proposed design in the revised version.

**“**The proposed antenna has simple structure, easy to design, fabricate and deploy on human body and has important significance in scaling up this design to generate multiple OAM modes for carrying multiple signals simultaneously”

**Comment 5: Introduction section can be improved by adding more discussions on various methods to realize high performance antennas such as metamaterial and metasurface approaches. Below are helpful suggestions.**

**Author’s response:** Thank you for your feedback. Yes we have added some of the relevant references as suggested by the reviewer. The new references are [24]-[27].

[24]	Awan, W.A.; Naqvi, S.I.; Ali, W.A.E.; Hussain, N.; Iqbal, A.; Tran, H.H.; Alibakhshikenari, M.; Limiti, E. Design and Realization of a Frequency Reconfigurable Antenna with Wide, Dual, and Single-Band Operations for Compact Sized Wireless Applications. Electronics 2021, 10, 1321.

[25]	M. Alibakhshikenari et al., "Impedance Bandwidth Improvement of a Planar Antenna Based on Metamaterial-Inspired T-Matching Network," in IEEE Access, vol. 9, pp. 67916-67927, 2021.

[26]	Iqbal, A.; Altaf, A.; Abdullah, M.; Alibakhshikenari, M.; Limiti, E.; Kim, S. Modified U-Shaped Resonator as Decoupling Structure in MIMO Antenna. Electronics 2020, 9, 1321.

[27]	Alibakhshikenari, M.; Virdee, B.S.; Shukla, P.; See, C.H.; Abd-Alhameed, R.; Khalily, M.; Falcone, F.; Limiti, E. Antenna Mutual Coupling Suppression Over Wideband Using Embedded Periphery Slot for Antenna Arrays. Electronics 2018, 7, 198.

**Comment 6: The feeding mechanism of the array antenna should be mentioned in detail.**

**Author’s response:** Thank you for your insightful comment. We have revised it and added the details of the feeding mechanism in Section 2.1.

**“**A carefully designed corporate feed network with the T-junction method was implemented to ensure that fed signal arrives at the patches with equal amplitude and desired phase differences. The combination of 50 Ω and 100 Ω feeding network was used to make sure that each radiating elements is receiving equal or almost same power amplitude to generate OAM waves with desired mode number”

**Comment 7: Why have the authors made some cuts at the corner of each patch? The impact of this method should be elaborated in depth.**

**Authors response:** Thank you for mentioning this concern. We have given a detailed explanation in Section 2.1 of the revised manuscript.

“Inset feed technique was used by cutting the two sides of feedline of each element to make sure there is good impedance matching between the transmission line and the patches so that maximum power reaches the patches through the feedline network.”

**Comment 8: Why are the meandered lines connecting the patches not symmetric?**

**Authors response:** Thank you for highlighting this issue. As we have shown in Figure 1 (c) and explained in section 2.1, the radiating elements including the feedlines/meandered lines were cut manually by hand hence there was an asymmetric lines due to human error. This can be avoided by cutting the feedlines more carefully or a cutting matching. As it can be seen from Figure 1 (a) that the meander lines connecting to Patches 3 and 4 are the mirror image of the meander line connecting to the Patches 1 and 2. Hence, the same feeding network of patches 1 and 2 was used for patches 3 and 4 which gave a 180° phase rotation. To generate OAM mode 1, the phase difference between the each element should be 90° and from patch 1 to patch 4 will be in the sequence of 0°, 90°, 180° and 270°. Our obtained result is very close with the theoretical design as it can be seen from Figure 2a. The phase increment from patch 1 to patch 4 of our design is 2°, 91°, 182° and 273° with maximum percentage error 1.11%.

“Fig 1 (C) provides a summary of the proposed antenna's fabrication process. First, a piece of paper with the proposed antenna's structure printed with its exact dimensions from the simulation was used as a guide to cut the super-Shieldit electro-textile. Gerber software was used in this stage. The radiating patches and feedline won't bend or shift while being attached to the substrate, which is another benefit of printing the structure with the proper dimensions. In order to physically cut the printed paper with a cutter and scissors, it was tightly fastened to a piece of super-Shieldit fabric. Next, the substrate was cut per the simulated design dimension, and the patches and feedlines were carefully placed on top of the substrate. To excite the antenna, a 50 Ω female Sub Miniature version A (SMA) connector was soldered onto the feedline. And lastly, a multimeter was used to check the continuity between the SMA connector inner pin and the feedline.”

**Comment 9: Is it possible to apply the proposed method to implement an array antenna with a higher number of radiation elements?**

**Authors response**: Thank you for your constructive query. Yes, it is possible to implement an array with higher number of radiaiting elements using the proposed concept. This paper focused on generating OAM mode +1 hence four elements were enough. However, for higher number of modes such as 2, 3, 4 or so on, higher number of radiating elements are required. If other researchers want to generate higher modes, they would have to use more radiating elements. Still, the proposed concept can be used undoubtedly. We have covered this concern in one of our review paper “S. K. Noor et al., "A Review of Orbital Angular Momentum Vortex Waves for the Next Generation Wireless Communications," in IEEE Access, vol. 10, pp. 89465-89484, 2022, doi: 10.1109/ACCESS.2022.3197653”

**Comment 10: How have authors reduced the mutual coupling between the radiation elements which is an important parameter to realize array antennas? Below are some helpful methods to improve the isolation between the array elements which can be briefly mentioned in the introduction section.**

**Authors response**: Thank you for your feedback. Yes we have added some of the relevant references as suggested by the reviewer. The new references are [24]-[27].

[24]	Awan, W.A.; Naqvi, S.I.; Ali, W.A.E.; Hussain, N.; Iqbal, A.; Tran, H.H.; Alibakhshikenari, M.; Limiti, E. Design and Realization of a Frequency Reconfigurable Antenna with Wide, Dual, and Single-Band Operations for Compact Sized Wireless Applications. Electronics 2021, 10, 1321.

[25]	M. Alibakhshikenari et al., "Impedance Bandwidth Improvement of a Planar Antenna Based on Metamaterial-Inspired T-Matching Network," in IEEE Access, vol. 9, pp. 67916-67927, 2021.

[26]	Iqbal, A.; Altaf, A.; Abdullah, M.; Alibakhshikenari, M.; Limiti, E.; Kim, S. Modified U-Shaped Resonator as Decoupling Structure in MIMO Antenna. Electronics 2020, 9, 1321.

[27]	Alibakhshikenari, M.; Virdee, B.S.; Shukla, P.; See, C.H.; Abd-Alhameed, R.; Khalily, M.; Falcone, F.; Limiti, E. Antenna Mutual Coupling Suppression Over Wideband Using Embedded Periphery Slot for Antenna Arrays. Electronics 2018, 7, 198.

**Reviewer #3:**

**Comment 1. Author incorporates all the suggested reviews and now I believe it’s ready to accept.they also update the grammatical mistakes too.**

**Authors response:** Thank you so much for accepting and acknowledging our previous revisions. We have corrected the grammatical mistakes in the revised version.

**Reviewer #4:**

**This manuscript has found suitable conditions compared to the previous version. Therefore, it can be published in this magazine. However, it is recommended that authors carefully review the entire text of the manuscript for spelling and grammar.**

**Authors response:** Thank you so much for accepting and acknowledging our previous revisions. We have corrected the grammatical mistakes in the revised version.

**Reviewer #5:**

**Comment 1: There is no mention about the polarization of the proposed design in the manuscript.**

Authors response: Thank you for your valuable concern. In OAM waves, The OAM is related to the spatial phase distribution of the EM wave. OAM is carried by an EM wave with the azimuthal phase distribution of exp (ilφ), and such an EM wave is termed an OAM wave. Therefore, the polarization of OAM waves is in helical shape along the direction of propagation where the phase changes by 2πl. Therefore, it is not linearly polarized nor circularly polarized rather it’s a whole new different type of polarization. We have covered this concern in one of our review paper “S. K. Noor et al., "A Review of Orbital Angular Momentum Vortex Waves for the Next Generation Wireless Communications," in IEEE Access, vol. 10, pp. 89465-89484, 2022, doi: 10.1109/ACCESS.2022.3197653”.

**Comment 2: There are several grammatical errors in the manuscript.**

**Authors response:** Thank you for highlighting this issue. We have corrected the manuscript’s grammatical errors in the revised version.

**Comment 3: The authors in the manuscript don’t mention whether the results like radiation pattern, gain, bandwidth etc. achieved are on-body or free space results. And if there is any difference between the two the authors are encouraged to showcase the same.**

**Authors response:** Thank you for constructive comment and concern. Parameters such as radiation pattern and gain are achieved for free space results. However, as shown in Figure 5 (b), the reflection coefficient (S_11_) and bandwidth was measured On-body as well. It was observed that, when the antenna was placed on body, the frequency of operation moved to higher bands however it still covered the desired frequency range. This shifting of frequency is called the Detuning effect. There is shifting in frequency when placed on human body because of the changes in the antenna’s internal impedance, Zin when it was placed on a material in our case the human body having different dielectric properties. Furthermore, human flesh has high conductivity (σ) and different dielectric properties as compared to a free space environment, thus this might have influenced its performance.

We have added this explanation in the revised manuscript.

“Besides, the antenna's internal impedance varies when it is placed on a material—in this case, the human body—that has varying dielectric characteristics, causing a shifting in frequency. This effect is called detuning effect. Moreover, the dielectric characteristics of human flesh differ from those of a free space environment and have a high conductivity (σ), which could have caused the shifting of frequency.”

**Comment 4: Is there any effect of body on the generation of OAM waves when the antenna is mounted on the body? And if yes, discuss in the manuscript.**

**Authors response:** Thank you for constructive comment and highlighting this point. In our research, we have analyzed the influence of bending on OAM waves generation however we haven’t studied the influence of human body on the generation of OAM waves. This can be one of our future study.
